# Stress-controlled decomposition routes in cubic AlCrN films assessed by *in-situ* high-temperature high-energy grazing incidence transmission X-ray diffraction

**DOI:** 10.1038/s41598-019-54307-7

**Published:** 2019-12-02

**Authors:** M. Meindlhumer, S. Klima, N. Jäger, A. Stark, H. Hruby, C. Mitterer, J. Keckes, R. Daniel

**Affiliations:** 1 Christian Doppler Laboratory for Advanced Synthesis of Novel Multifunctional Coatings at the Department of Materials Science, Montanuniversität Leoben, Leoben, Austria; 2 Helmholtz-Zentrum Geesthacht, Centre for Materials and Coastal Research, Geesthacht, Germany; 3 Voestalpine eifeler Vacotec GmbH, Düsseldorf, Germany; 4 Department of Materials Science, Montanuniversität Leoben, Leoben, Austria

**Keywords:** Materials science, Characterization and analytical techniques

## Abstract

The dependence of decomposition routes on intrinsic microstructure and stress in nanocrystalline transition metal nitrides is not yet fully understood. In this contribution, three Al_0.7_Cr_0.3_N thin films with residual stress magnitudes of −3510, −4660 and −5930 MPa in the as-deposited state were *in-situ* characterized in the range of 25–1100 °C using *in-situ* synchrotron high-temperature high-energy grazing-incidence-transmission X-ray diffraction and temperature evolutions of phases, coefficients of thermal expansion, structural defects, texture as well as residual, thermal and intrinsic stresses were evaluated. The multi-parameter experimental data indicate a complex intrinsic stress and phase changes governed by a microstructure recovery and phase transformations taking place above the deposition temperature. Though the decomposition temperatures of metastable cubic Al_0.7_Cr_0.3_N phase in the range of 698–914 °C are inversely proportional to the magnitudes of deposition temperatures, the decomposition process itself starts at the same stress level of ~−4300 MPa in all three films. This phenomenon indicates that the particular compressive stress level functions as an energy threshold at which the diffusion driven formation of hexagonal Al(Cr)N phase is initiated, provided sufficient temperature is applied. In summary, the unique synchrotron experimental setup indicated that residual stresses play a decisive role in the decomposition routes of nanocrystalline transition metal nitrides.

## Introduction

Protective nanocrystalline thin films prepared by physical vapour deposition are characterized by complex temperature-dependent microstructure/strain evolution and multistage decomposition routes, which control their physical and functional properties. Typical materials are transition metal nitride hard films based on Ti_x_Al_1−x_N and Cr_x_Al_1−x_N, which have been extensively studied in the past due to their beneficial functional properties^1^. A special interest has been devoted, in particular, to AlCrN because of its ability to form a metastable solid solution by replacing Cr over a wide concentration range with Al in the cubic (c) B1 structure, resulting in enhancement of mechanical properties, wear and especially oxidation resistance compared to TiAlN^2^. However, when the solubility limit of AlN in CrN is exceeded, stable wurtzite (w) B4 Al(Cr)N is formed^3,4^. w-Al(Cr)N forms in several steps also when c-AlCrN solid solution is annealed at temperatures above ~800 °C. Firstly, w-Al(Cr)N precipitates are formed at the grain boundaries (GBs), followed by the formation of h-Cr(Al)N_0.5_-phase as a consequence of nitrogen loss with a subsequent two-step decomposition of CrN into Cr_2_N and Cr^5–8^. The determination of the onset temperature of the phase decomposition (*T*_o,d_) over a wide Al composition range, as well as its correlation to the film microstructure and intrinsic stress, is crucial because the formation of the stable w-Al(Cr)N phase results in a reduction of hardness, wear and oxidation resistance^9–13^. In the case of AlCrN-based thin films, however, the complex interplay between temperature-dependent characteristics and properties such as the onset of phase transformation, coefficients of thermal expansion (CTEs), gradients of residual stresses, defect density, hardness, toughness and elastic modulus in different atmospheres is however not yet fully understood^5,6,8,14–16^. Here, especially the role of microstructure and residual stresses has not been evaluated thoroughly.

In the case of transition metal nitride hard thin films, *in-situ* synchrotron XRD has been used to study temperature-dependent phenomena and physical parameters like phase evolution, lattice parameters and/or in-plane strains (i) in powders of TiAlN and TiCrAlN films and (ii) in thin slices of TiAlN and TiZrAlN films in transmission diffraction geometry^17–21^. The former experiments concentrated mostly on the understanding of complex decomposition routes and related lattice parameter/strain changes.

The motivation of this work is to further extend the possibilities of *in-situ* XRD characterization, which will be used to obtain a complex picture of the multistage decomposition routes and also microstructure/strain changes in AlCrN-based thin films during thermal cycling. We use a newly developed *in-situ* high-temperature high-energy grazing incidence transmission synchrotron X-ray diffraction (HT-HE-GIT-XRD)^22^ to simultaneously characterize temperature evolution of (i) phases, (ii) residual stresses, (iii) thermal strains, (iv) CTEs, (v) domain sizes and (vi) texture up to 1100 °C. Primarily, the onset temperature of the decomposition of metastable c-AlCrN into stable c-Cr(Al)N and w-Al(Cr)N phases has been investigated as a function of the as-deposited residual stress state and microstructure, intentionally predefined by the applied deposition conditions. The multi-parameter temperature-dependent structure-property correlations indicate a decisive role of residual stress magnitude in the decomposition routes of metastable c-AlCrN.

## Results

### *In-Situ* phase analysis

**Figure 1:**
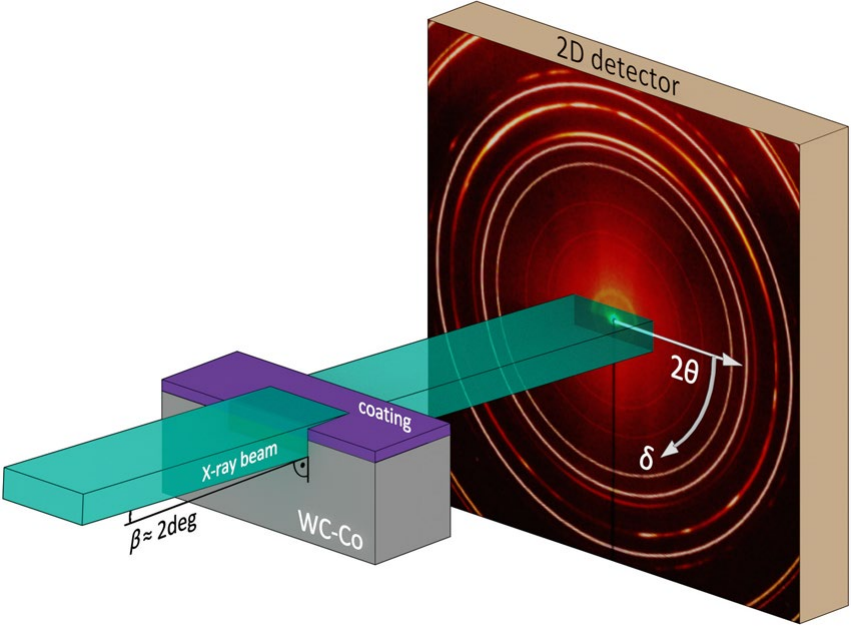
Schematic representation of the *in-situ* high-temperature high-energy grazing incidence transmission X-ray diffraction method (HT-HE-GIT-XRD).

Thermal stability of the metastable AlCrN thin films was investigated in the temperature range between room temperature (RT) and 1100 °C by analysing Debye-Scherrer rings for the detector azimuthal angles *δ* in the range of 0 to −180 deg by a sectoral integration of the patterns, as the rest of the data, corresponding to the *δ* complementary angles, comprised mostly diffraction signal from the WC-Co substrate (cf. Fig. 1).

**Figure 2:**
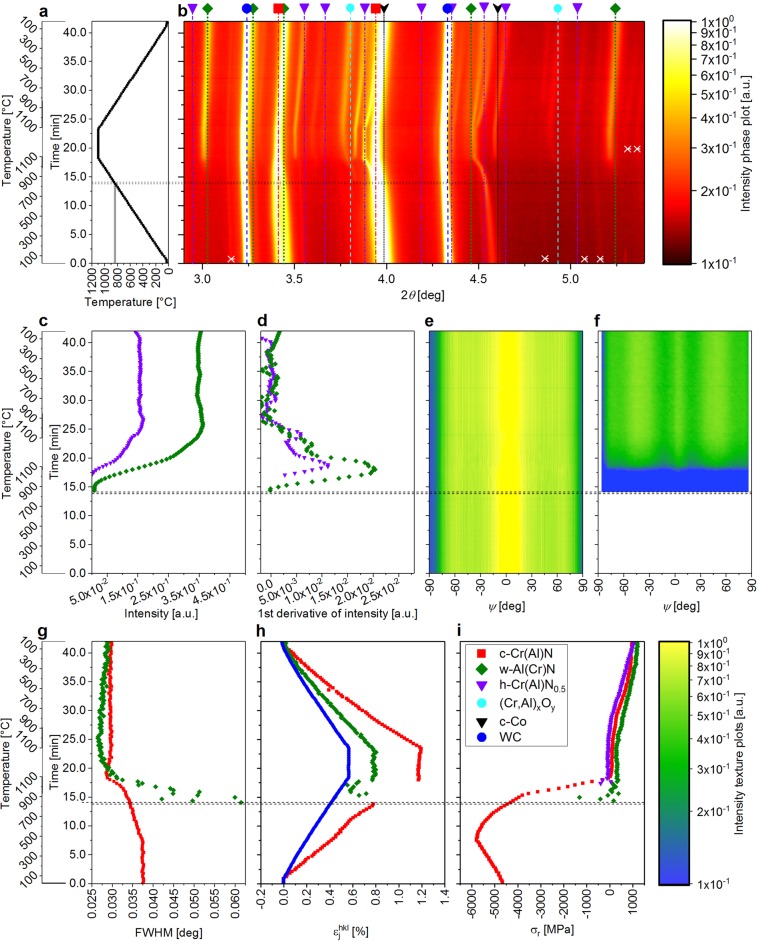
The applied temperature cycle (**a**) and the experimental data of the AlCrN film B, phase plot with indicated diffraction angles for tabulated particular phases, where the white crosses indicate additional diffraction peaks representing 2^nd^ order diffraction due to the presence of the second harmonic’s wavelength in the primary beam (**b**), evolution of intensity (**c**) and the 1^st^ derivative of the intensity (**d**) of w-Al(Cr)N 100 (green) and h-Cr_2_N 100 (violet) reflections, the texture plot for c-Cr(Al)N 111 reflection indicating 〈111〉 fibre texture (**e**), texture plot for w-Al(Cr)N 100 reflection indicating overlapping 〈100〉 and 〈110〉 fibre texture (**f**), evolution of FWHM of c-Cr(Al)N 111 (red) and w-Al(Cr)N 100 (green) reflections (**g**), thermal expansion of c-Cr(Al)N (red, evaluated from the 200 reflection), w-Al(Cr)N (green, 100 reflection) and WC substrate (blue) (**h**) and residual stress evaluated from the c-Cr(Al)N 200 (red), w-Al(Cr)N 100 (green) and h-Cr_2_N 100 (violet) reflections (**i**).

**Figure 3:**
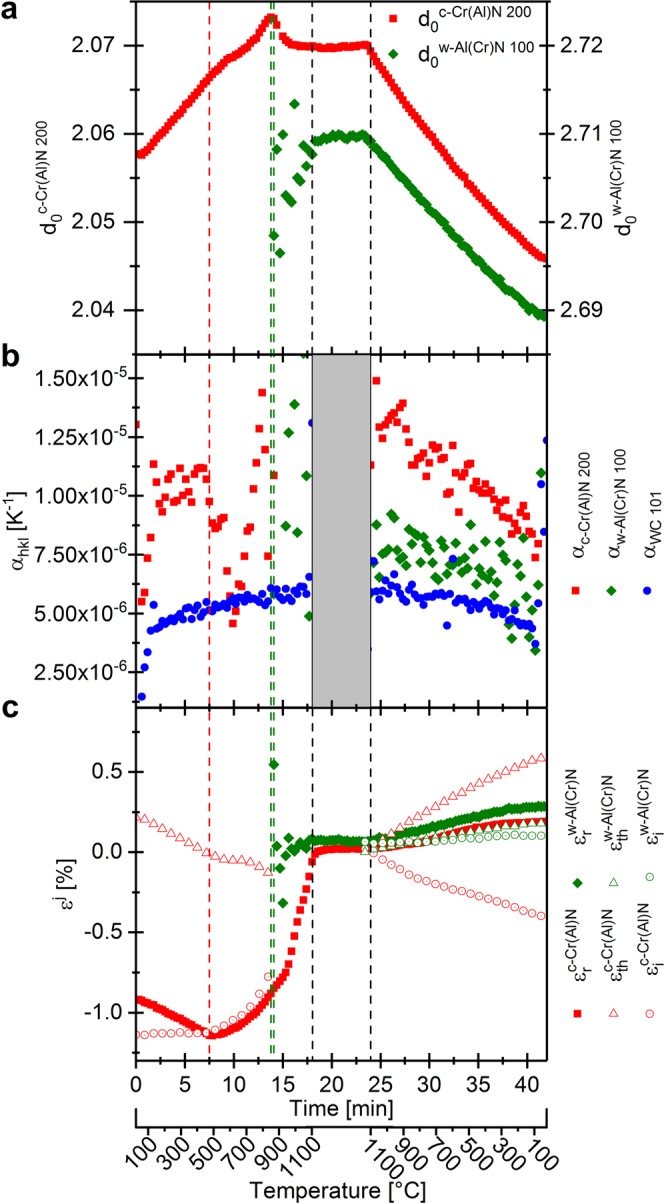
Experimental assessment for film B deposited at 400 °C: the development of the unstrained lattice parameter *d*_0_(T) of c-Cr(Al)N and w-Al(Cr)N phase over the temperature (**a**), the thermal expansion coefficient calculated for the individual reflections (**b**) and the evolution of thermal, intrinsic and residual strain over the temperature cycle for c-Cr(Al)N (red) and w-Al(Cr)N (green), respectively (**c**). The vertical red dashed line represents the deposition temperature, the green dashed line the onset of the phase decomposition and the vertical black segmented lines the beginning and the end of the holding segment.

Along the text, mainly experimental data from the film B are presented and interpreted in Figs. 2 and 3, whereby the results from other two films can be found in the supplementary Data. The phase plots displayed in Fig. 2b and in Suppl. Figs. 1b and 3b represent temperature-dependent evolutions of diffraction intensities in the 2*θ* range of 2.9 to 5.4 deg. For every Bragg’s angle *θ* (and annealing temperature *T*), the particular intensity was obtained by integrating the diffraction signal from the detector in the azimuthal angle range *δ* from −180 to 0 deg. The temperature and phase evolution during one temperature cycle are presented in Fig. 2a,b together with the tabulated reflection positions of w-AlN, c-CrN, Cr_2_N and substrate WC and c-Co phases adapted from the JCPDF database^23^. The featured 111 and 200 reflections indicate that the Al_0.7_Cr_0.3_N thin film deposited at T_S, B_ = 400 °C was at RT in metastable state and possessed the face-centred cubic B1 (c) structure. In addition to the reflections emanating from the film, also WC 100 and 101, as well as c-Co 111 and 200 reflections corresponding to the cemented carbide substrate are visible (Fig. 2b). With the temperature increase, all diffraction peaks shift to smaller diffraction angles, as a consequence of thermal expansion of the film and substrate phases and changes in thin film stress state. At temperatures above ~830 °C, the metastable c-Al_0.7_Cr_0.3_N phase started to decompose into c-Cr(Al)N and w-AlCrN phases, as indicated by the presence of 100 diffraction peak (at 2*θ* = 3.026 deg) from the w-AlCrN phase detected above that critical temperature. This process corresponds to the formation of Al(Cr)N precipitates at the GBs with wurtzite crystallographic structure, as extensively discussed elsewhere^5,6^. For the films A, B and C, the different onset temperatures of the phase decomposition were determined as 698–748 °C, 834–851 °C and 880–914 °C (Fig. 5c), respectively. The uncertainty of ~20–50 °C originates from the nonzero detector exposure time and the relatively high heating rate.

The w-Al(Cr)N 100 diffraction peak was selected to semi-quantitatively assess the progress of the decomposition process of the metastable cubic AlCrN phase and the formation of the w-AlCrN polytype. The variation in the intensity of the w-Al(Cr)N 100 reflection and its first derivative are depicted in Fig. 2c,d, respectively. Since the decomposition of the metastable c-AlCrN is a diffusion-controlled process, its end can be identified by the decrease in the first derivative of the w-Al(Cr)N 100 reflection intensity to zero (${\rm{\partial }}{I}_{{\mathtt{w}}{\mathtt{-}}{\mathtt{A}}{\mathtt{l}}{\mathtt{C}}{\mathtt{r}}{\mathtt{N}}{\mathtt{100}}}(T)/{\rm{\partial }}T\to 0$) (Fig. 2d).

After the onset of the decomposition of c-AlCrN to c-Cr(Al)N and w-Al(Cr)N, non-stoichiometric (Cr,Al)_x_O_y_ is formed as a consequence of residual oxygen presence in the dilatometer chamber. The (Cr,Al)_x_O_y_ stoichiometry changes throughout further heating and the holding segment are indicated by the changes of the 2*θ*-positions of the respective reflections in Fig. 2b. The formation of (Cr,Al)_x_O_y_ is in agreement with the literature data, where previous results stated reduced oxidation resistance of c-Cr(Al)N and w-Al(Cr)N phases after decomposition^9,10^.

In Fig. 2b, the onset of the decomposition of CrN into Cr_2_N can be also identified by the occurence of the Cr_2_N reflections from several Cr_2_N crystalline polytypes at the temperature of ~1080 °C, in agreement with literature values^6,7,24^. No further crystallographic changes were found during cooling down to RT.

### *In-Situ* qualitative texture analysis

Qualitative texture analysis was carried out by a radial (*θ*) integration of Debye-Scherrer rings providing azimuthal intensity distributions *I*^hkl^(*δ*, *T*) and the data were plotted as a function of the *ψ* angle as *I*^hkl^(*ψ*, *T*), where *ψ* represents the angle between the film normal and the diffraction vector. Due to the high energy of photons resulting in negligibly diffraction angles (cf. Fig. 2b), the transformation between the azimuthal angle *δ* and the tilt angle *ψ* can be generally expressed as

1 $$\cos \,\psi =\,\sin \,\delta \cdot \,\cos \,\theta \cong \,\sin \,\delta $$

and further

2 $$\psi =\delta +90,$$

as shown by Keckes *et. al*.^25^.

A qualitative texture analysis was carried out for the c-AlCrN 111 and w-AlCrN 100 reflections. Therefore, the azimuthal intensity distributions *I*^hkl^(*ψ*, *T*) are displayed in Fig. 2e,f for c-Cr(Al)N 111 and w-Al(Cr)N 100 Debye-Scherrer rings, respectively. The diffraction intensities recorded at *ψ* = 0 and ±90 degrees correspond to the out-of-plane and in-plane orientations of the diffraction vectors, respectively. The plots in Fig. 2e,f allow to draw conclusions about the preferred orientation of the fibre-textured phases. It revealed the 〈111〉 fibre texture of the cubic c-Cr(Al)N phase, as the maximum intensity of the c-Cr(Al)N 111 reflection was found approximately at the diffraction vector orientation which is parallel to the film normal and corresponds to $\psi \cong 0\,{\rm{\deg }}$ (cf. exemplary data for the film B in Fig. 2e and Supplementary Figs. S1e and S3e). Texture analysis carried out for the w-Al(Cr)N 100 reflection revealed azimuthal maxima at $\psi \cong 0,\,\pm 60\,{\rm{\deg }}$ and $\psi \cong \pm 30\,{\rm{\deg }}$ corresponding to 〈100〉 and 〈110〉 fibre textures, respectively (Fig. 2f, and Supplementary Figs. S1f and S3f for A and C samples). This indicates that the densely packed {002} planes of the wurtzite phase were oriented actually parallel to the columnar GBs of the c-Cr(Al)N phase^26^. The temperature dependencies of *I*^hkl^(*ψ*, *T*) for the c-Cr(Al)N 111 and w-Al(Cr)N 100 reflections in Fig. 2e,f indicate that the textures of the particular phases did not change significantly during the thermal cycles.

### Full Width at half maximum analysis

Generally, the full width at half maximum (FWHM) of X-ray diffraction peaks correlates with the size of coherently diffracting domains as well as with the density of structural defects such as dislocations and lattice distortions, represented by strains of 2^nd^ and 3^rd^ order. Since the AlCrN crystallites of all thin films exhibited columnar grain morphology, which overall did not change during the thermal treatment, it can be assumed that the FWHM changes over the temperature cycle are sensitive primarily to the variation of structural defect density in the nanocrystals^25,27–29^. In order to evaluate FWHM, c-Cr(Al)N 111 and w-Al(Cr)N 100 diffraction peaks were fitted using the Pseudo-Voigt function for the diffraction vector out-of-plane orientation. The results in Fig. 2g indicate that the defect density in c-CrAlN remains constant up to the deposition temperature of 400 °C, below which no relaxation processes took place. Above ~400 °C, a relaxation of structural defects^30^ is reflected by a continuous decrease in the FWHM. A further decrease of the FWHM of c-CrAlN above the onset temperature of the phase decomposition between ~850 and ~1100 °C (Fig. 2g) indicates a gradual defect recovery accompanied by micro-strain development as the w-Al(Cr)N phase is formed in the film. On the contrary, a slight increase in the FWHM was detected during the subsequent temperature holding segment, which is attributed to the ongoing phase decomposition and a possible reduction of the size of the coherently diffracting domains due to the emerging of the w-Al(Cr)N and h-Cr(Al)N_0.5_ phase fractions.

The corresponding FWHM of w-Al(Cr)N 100 reflection was relatively large at the beginning of the phase decomposition (Fig. 2g). In the next heating steps and during the holding segment, the FWHM continuously decreases, which reflects the ongoing phase transformation accompanied by the w-Al(Cr)N phase crystallite growth. On the contrary, no significant change in the FWHM was detected during the cooling segment of the temperature cycle.

**Figure 4:**
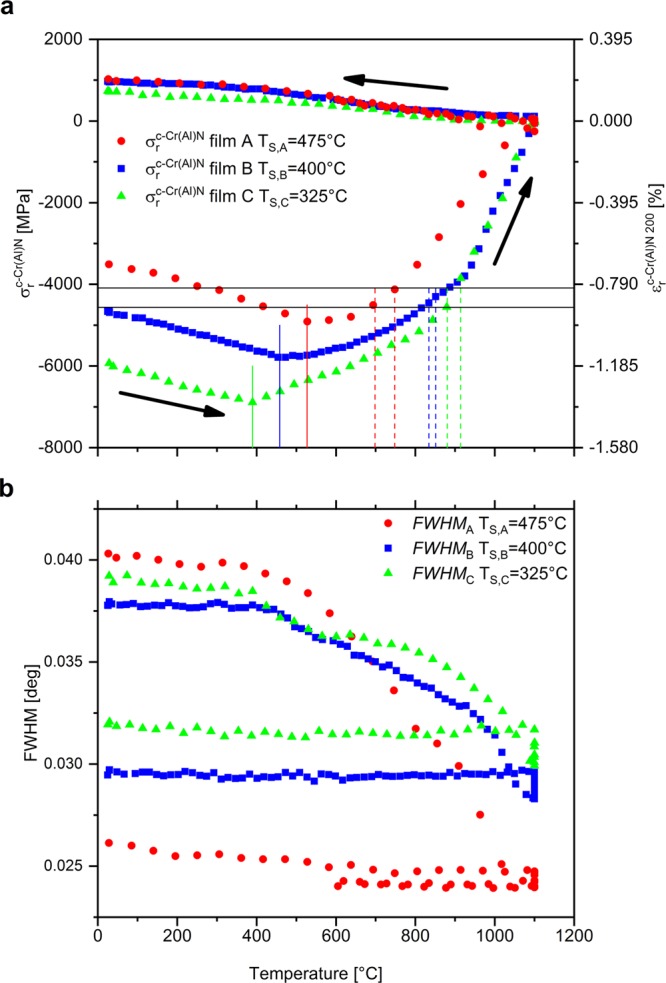
Residual stress evolutions over the heating cycles for films A, B and C deposited at 475 °C, 400 °C and 325 °C,respectively (**a**). Full vertical lines indicate the end of the thermoelastic regime, dashed lines indicate the onset of phase decomposition and horizontal lines show the mean values of stress magnitude for the three onsets of phase decomposition in the individual coatings. FWHM of c-AlCrN evolution up to 1100 °C for coatings A, B and C deposited at 475 °C, 400 °C and 325 °C, respectively (**b**).

The temperature dependent behaviour of FWHM was similar for all films, as shown in Fig. 4b. The differences in the magnitudes and temperature dependencies of the FWHM, which correlate with the phase microstructural evolution during thermal cycling, can be attributed to the specific as-deposited states in terms of microstructure and residual stress.

### Unstrained lattice parameter analysis

The changes in the temperature-dependent unstressed lattice parameters ${d}_{0}^{hkl}(T)$ of the particular phases were evaluated using Bragg’s law according to

3 $${d}_{0}^{hkl}({\delta }^{\ast })=\frac{\lambda \,}{2\,\sin \,{\theta }^{hkl}({\delta }^{\ast })},$$

where *λ* is the X-ray wavelength and *θ*(δ^*^) is the diffraction angle of the selected Debye-Scherrer ring at the ring azimuthal orientation δ^*^. The diffraction signal collected at the ring position δ* represents the diffraction on {*hkl*} crystallographic planes, which are, due to the equibiaxial in-plane stress state, not strained. This azimuthal angle δ* can be determined as follows^29,31^

4 $$\sin \,{\delta }^{\ast }=\sqrt{\frac{1-{\nu }^{hkl}}{1+{\nu }^{hkl}}}.$$

here, *ν*^hkl^ is the Poisson’s ratio of the investigated Debye-Scherrer ring *hkl* of the phase of interest. Poisson’s ratios *ν*^200^ and *ν*^100^ of 0.132 and 0.253, calculated from the X-ray elastic constants using the Hill-type grain interaction model^32^, were used to determine the stress-free lattice parameters ${d}_{0}^{hkl}({\delta }^{\ast })$ of the c-AlCrN and w-Al(Cr)N phases, respectively^33,34^. The unstrained sample orientations of δ*_c_ = ~−61 and δ*_w_ = ~−51 deg were found for c-Cr(Al)N 200 and w-Al(Cr)N 100 rings, respectively. The WC 101 Debye-Scherrer rings were found to be unaffected by the presence of residual stress in the film.

Using Eqs. (3) and (4), lattice spacing of WC at RT was evaluated from the WC 101 reflection as 1.88126 Å, which is ~0.1% smaller than the tabulated lattice parameter^23^ of 1.88266 Å, which can be attributed to the limits in the detector calibration accuracy. For the cubic AlCrN phase, two different lattice parameters *d*_0_^200^ of 2.05838 Å for the as-deposited state and 2.04584 Å for the annealed state were obtained. In the case of the w-Al(Cr)N phase, a lattice parameter *d*_0_^100^ of 2.68915 Å after the thermal cycle was determined, which is close to the tabulated lattice parameter^23^ of 2.69542 Å.

Temperature dependencies of unstressed lattice parameter *d*_0_^200^(*T*) of the c-Cr(Al)N phase and *d*_0_^100^(*T*) of the w-Al(Cr)N are presented in Fig. 3a. The scattering of the lattice parameter *d*_0_^100^(*T*) was caused by the small measurable volume of the w-Al(Cr)N crystallites at early stages of the decomposition. The relative changes in the lattice parameters ${d}_{0}^{{\rm{hkl}}}(T)$ of the particular (*j*) phases with respect to RT ($\frac{{d}_{0}^{{\rm{hkl}}}(T)}{{d}_{0,\,\mathrm{RT}}^{{\rm{hkl}}}}$) were used to quantify the thermal expansion ${\varepsilon }_{{\rm{j}}}^{{\rm{hkl}}}(T)$ as follows

5 $${\varepsilon }_{j}^{hkl}(T)=\frac{{d}_{0}^{hkl}(T)-{d}_{0,RT}^{hkl}}{{d}_{0,\,RT}^{hkl}}.$$

In Fig. 2h, the different slopes of the ${\varepsilon }_{{\rm{j}}}^{{\rm{hkl}}}(T)$ dependencies for c-Cr(Al)N, w-Al(Cr)N and WC phases indicate differences in the CTEs. In the case of WC the thermal expansion of ${\varepsilon }_{{\rm{WC}}}^{101}(T)$ ~0.6% at 1100 °C is in good agreement to values of 0.2% after heating to 400 °C reported by Hidnert^35^.

The variation of the lattice parameter during heat treatment is a consequence of (i) thermal expansion of the particular crystal lattices, (ii) defect annihilation and lattice recovery, (iii) phase decomposition and diffusion of Al out of the cubic Cr(Al)N phase and (iv) nitrogen loss and subsequent formation of Cr_2_N at elevated temperatures, both accompanied by the massive formation of point defects within the cubic phase.

### Experimental thermal expansion coefficients and thermal strains

The evaluated ${d}_{0}^{{\rm{hkl}}}(T)$ dependencies were used to determine (i) the CTEs *α*_hkl_ of the film and substrate phases and, subsequently, (ii) the experimental in-plane thermal strain ${\varepsilon }_{{\rm{th}}}^{{\rm{j}}}$ formed within individual phases (*j*) during temperature changes (Eqs. (5) and (6)). CTEs were determined as follows

6 $${\alpha }_{{\rm{hkl}}}(T)=\frac{1}{{d}_{0}^{{\rm{hkl}}}(T)}\frac{\partial {d}_{0}^{{\rm{hkl}}}(T)}{\partial T}\cong \frac{\partial {\varepsilon }_{{\rm{j}}}^{{\rm{hkl}}}(T)}{\partial T},$$

where the ${d}_{0}^{{\rm{hkl}}}(T)$ is the unstrained lattice parameter. The *hkl* subscript was used in ${\alpha }_{{\rm{hkl}}}(T)$ only to denote the Debye-Scherer ring, which was used to evaluate the particular CTE for the individual phase.

The CTEs determined for the c-Cr(Al)N and w-Al(Cr)N phases of the film B over both the heating and cooling segments are shown in Fig. 3b and represent actually the first derivation of the *d*(*T*) dependencies from Fig. 3a with respect to the temperature *T*. The relatively large scattering of the CTEs experimental data is caused by the small intensity of the diffraction signal as well as small lattice parameter changes in the range of ~1%. The microstructural processes responsible for the non-linear temperature evolution of CTEs will be extensively discussed in section 4.2. Quantitatively, both phases showed a typical development of their CTEs within the investigated temperature region, as Bartosik *et al*. showed for powdered AlCrN films^36^. For the WC substrate, values of the CTE in the range of ~4 × 10^–6^ K^−1^ at RT to ~6 × 10^−6^ K^−1^ at 1100 °C were determined, in agreement with Hidnert^35^.

Subsequently, experimental values of ${\alpha }_{{\rm{hkl}}}(T)$ from the substrate and the film were used to quantify changes in experimental in-plane thermal strains $\Delta {\varepsilon }_{{\rm{th}}}^{{\rm{j}}}(T)$ developed in the film during the particular heating and cooling segments^37^ as follows

7 $$\Delta {\varepsilon }_{{\rm{th}}}^{{\rm{j}}}(T)=\int ({\alpha }_{{\rm{s}}}(T)-{\alpha }_{{\rm{hkl}}}(T))dT,$$

where *α*_hkl_ and *α*_S_ are the experimental CTEs of the film and the substrate, respectively.

Since the thermal strain ${\varepsilon }_{{\rm{th}}}^{{\rm{c}}}(T)$ in the c-Cr(Al)N phase is assumed to be zero at the deposition temperature *T*_S_ (see the arguments in section *In-situ* Residual Strain and Stress Evolution), i.e.

8 $${\varepsilon }_{{\rm{th}}}^{{\rm{c}}}({T}_{{\rm{S}}})=0,$$

the determination of the accumulated in-plane thermal strain up to ~800 °C is rather straightforward and the results are shown in Fig. 3c. In order to evaluate the strain development during cooling down, an assumption was considered that in-plane residual strain is negligible during the holding segment at 1100 °C in both phases (Figs. 2i and 4a), which implies that in-plane thermal and intrinsic strains could also be neglected:

9 $${\varepsilon }_{{\rm{i}}}^{{\rm{hkl}}}({1100}^{\circ }C)={\varepsilon }_{{\rm{th}}}^{{\rm{hkl}}}({1100}^{\circ }C)\cong 0.$$

The formalism from Eq. (7) could thus be used to evaluate the temperature dependencies of $\Delta {\varepsilon }_{{\rm{th}}}^{{\rm{j}}}(T)$ from the data of Fig. 3b for both c-Cr(Al)N and w-Al(Cr)N phases, which are presented together with the in-plane residual strains in Fig. 3c.

### *In-situ* residual strain and stress evolution

In order to evaluate residual in-plane strain *ε*_r_^hkl^ and stress *σ*_r_, an azimuthal integration of the diffraction patterns was performed over the azimuthal angle *δ* range from 0 to −180 deg in Δ*δ* segments of 5 deg. Thus, 36 radial intensity distributions *I*(*θ*, *Δδ*, *T*) were obtained for each exposure. The positions of c-Cr(Al)N 200, w-Al(Cr)N 100 and WC 101 diffraction peaks *θ*^hkl^(Δ*δ*_*i*_,*T*) were determined by fitting the XRD patterns using a Pseudo-Voigt function. The equibiaxial in-plane stress *σ*_r_ can be determined according to

10 $$\frac{\partial {d}^{hkl}(\Delta \delta ,T)}{\partial sin^{2}\delta }={\sigma }_{r}(T)\frac{1}{2}{s}_{2}^{{\rm{hkl}}}\times {d}_{0},$$

where *d*^hkl^(Δ*δ*_i_, *T*) is the lattice parameter evaluated for a particular Δ*δ*_i_ segment, *s*_2_^hkl^ is the X-ray elastic constant and *d*_0_ is the unstrained lattice parameter. Since the temperature dependence of ½*s*_2_^hkl^ was neglected due to the missing high-temperature ½*s*_2_^hkl^ values, in-plane stresses are presented together with the evaluated in-plane residual strains in Fig. 4a. The residual biaxial in-plane X-ray elastic strain *ε*_r_^hkl^ can be calculated using the same approach as follows

11 $$\frac{\partial {d}^{{\rm{hkl}}}(\Delta \delta ,T)}{\partial sin^{2}\delta }={\varepsilon }_{{\rm{r}}}^{{\rm{hkl}}}(T)\frac{1+{\nu }^{{\rm{hkl}}}}{1-{\nu }^{{\rm{hkl}}}}\times {d}_{0},$$

but with the advantage, that all parameters are known except of the Poisson’s ratio *ν*^hkl^ of the thin film, which was adopted from literature.

The elastic constants^33^ ½*s*_2_^200^ of 0.2575 × 10^−5^ MPa^−1^ and *ν*^200^ of 0.132 were used for the c-AlCrN 200 reflection. The elastic strain in the wurtzite Al(Cr)N phase was determined by analysing the w-Al(Cr)N 100 reflection and by taking into account elastic constants^34^ ½*s*_2_ of 0.4052 × 10^−5^ MPa^−1^ and *ν*^100^ of 0.253. For h-Cr_2_N elastic constants^24^ ½*s*_2_ of 0.3713 × 10^−5^ MPa^−1^ and *ν*^100^ of 0.293 were used. All X-ray elastic constants were calculated using the Hill-type grain interaction model^32^.

Since ${\varepsilon }_{{\rm{r}}}^{{\rm{hkl}}}(T)$ values are *hkl* dependent, equibiaxial reflection-independent elastic strains ${\varepsilon }_{{\rm{r}}}^{{\rm{j}}}(T)$ can be calculated for the individual phases (*j*) using

12 $${\varepsilon }_{r}^{j}(T)=\frac{\frac{{E}^{{\rm{hkl}}}}{1-{\nu }^{{\rm{hkl}}}}}{\frac{E}{1-\nu }}{\varepsilon }_{{\rm{r}}}^{{\rm{hkl}}}(T),$$

where *E* is the Young’s modulus and *ν* is the Poisson’s ratio of the phase.

Figure 2i presents the evolution of the residual stress in the c-AlCrN and w-AlCrN phases. In Fig. 4a, the experimental data are shown as a function of the applied temperature for all films A, B and C, deposited at temperatures 475, 400 and 325 °C, respectively. By heating the sample from RT to the deposition temperature *T*_S_, the contribution of the thermal stress to *σ*_r_ decreases and *σ*_r_ at *T*_S_ thus corresponds exclusively to the intrinsic stress. The compressive stress state in the film B at RT (Figs. 2i and 4a) was −4660 MPa and increased with the annealing temperature to −5790 MPa at 400 °C (corresponding to the deposition temperature).

**Figure 5:**
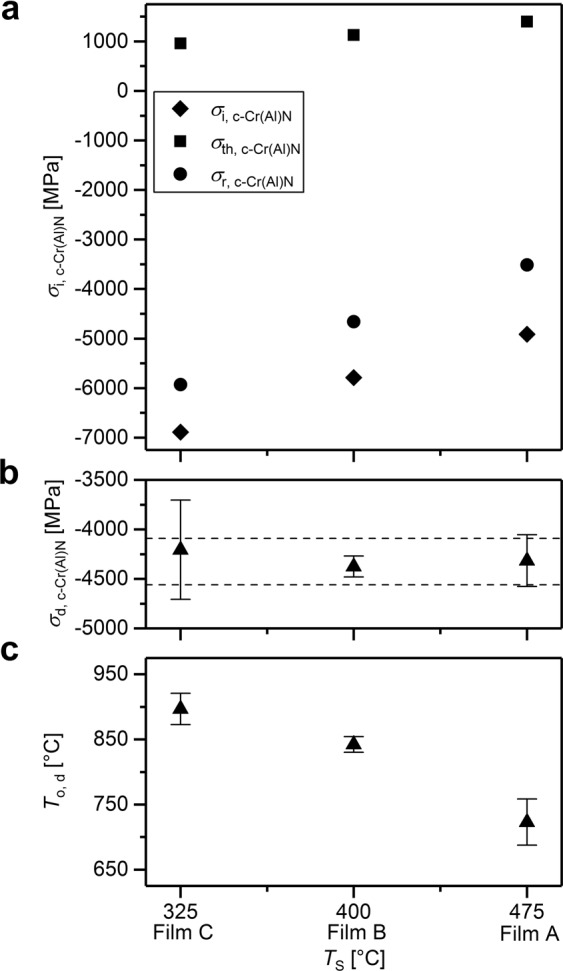
Correlations between applied deposition temperature *T*_S_ and (i) the intrinsic stress *σ*_i, c-CrN_, (ii) the thermal stress *σ*_th, c-CrN_, (iii) the resulting residual stress *σ*_r, c-CrN_ in the as-deposited state (**a**), (iv) the decomposition onset stress *σ*_d, c-CrN_ (**b**) and (v) the onset temperature of phase decomposition (**c**) *T*_o,d_ for films A (*T*_S_ = 475 °C), B (*T*_S_ = 400 °C) and C (*T*_S_ = 325 °C).

Magnitudes of residual stresses at RT, intrinsic and thermal stresses for all films are summarized in Fig. 5a. Remarkably, the mean *onset residual stress of the phase decomposition in all three films* was found between −4090 and −4500 MPa, as shown in Figs. 4a and 5b and Table 1, irrespective of the onset temperature of the phase decomposition derived from Fig. 5c (cf. also Sec. 4.2). Furthermore, although all three films were prepared with significantly different residual stresses, after the temperature cycle, the RT stresses in A, B and C films are with values of 1030, 970 and 740 MPa, respectively, comparable. This behaviour can be interpreted by the full stress relaxation at 1100 °C.

**Table 1:** Onset temperatures and onset stresses of phase decomposition for the films A, B and C.

	onset temperature of phase decomposition *T*_o,d_ [°C]			onset stress of phase decomposition *σ*_o,d_ [MPa]		
	A	B	C	A	B	C
lower limit	697	834	880	−4500	−4450	−4560
upper limit	748	851	914	−4130	−4300	−3850

### Experimental in-plane intrinsic strains

The residual equibiaxial in-plane stress *σ*_r_ in thin films prepared by physical vapour deposition originates from two dominant stress contributions, namely from intrinsic stress *σ*_i_, developed during ion-assisted film growth, and thermal stress *σ*_th_, developed during the cooling of the coated sample from the deposition temperature to RT as a consequence of the CTEs mismatch of the film and substrate^28^. Therefore, *σ*_r_ can be generally expressed as the sum of both of these stress components:

13 $${\sigma }_{{\rm{r}}}={\sigma }_{{\rm{i}}}+{\sigma }_{{\rm{th}}}.$$

Usually, only *σ*_r_ values can be determined using XRD and wafer curvature experiments. Here, we want to demonstrate that using our new multi-parameter approach, experimental temperature dependences of all three stress components from Eq. (13) can be evaluated. Due to the unique diffraction setup used in this study, the diffraction data from film and substrate could be simultaneously detected as a function of temperature and then used to evaluate equibiaxial residual strains in the film ${\varepsilon }_{r}^{j}$ (Sec. *In-situ* Residual Strain and Stress Evolution). Furthermore, by comparing the experimental CTEs of the thin film and the substrate, also equibiaxial experimental thermal strains ${\varepsilon }_{{\rm{th}}}^{{\rm{j}}}$ could be determined (Sec. Experimental Thermal Expansion Coefficients and Thermal Strains). Having both ${\varepsilon }_{{\rm{r}}}^{{\rm{j}}}$ and ${\varepsilon }_{{\rm{th}}}^{{\rm{j}}}$ components, ${\varepsilon }_{{\rm{r}}}^{{\rm{j}}}$ can be generally expressed as

14 $${\varepsilon }_{{\rm{r}}}^{{\rm{j}}}={\varepsilon }_{{\rm{i}}}^{{\rm{j}}}+{\varepsilon }_{{\rm{th}}}^{{\rm{j}}},$$

where ${\varepsilon }_{{\rm{i}}}^{{\rm{j}}}$ represents the unknown intrinsic strain. The intrinsic strain values can thus be calculated using Eqs. (7), (8), (11), (12) and (14). The temperature evolution of experimental residual, thermal and intrinsic strain ${\varepsilon }_{{\rm{i}}}^{{\rm{j}}}$(*T*) for the c-Cr(Al)N and w-Al(Cr)N phases are shown in Fig. 3c. The ${\varepsilon }_{{\rm{i}}}^{{\rm{j}}}$(*T*) data indicate that intrinsic strains start to change above the deposition temperature due the microstructure recovery and the subsequent phase transformation.

### Complementary analyses

**Figure 6:**
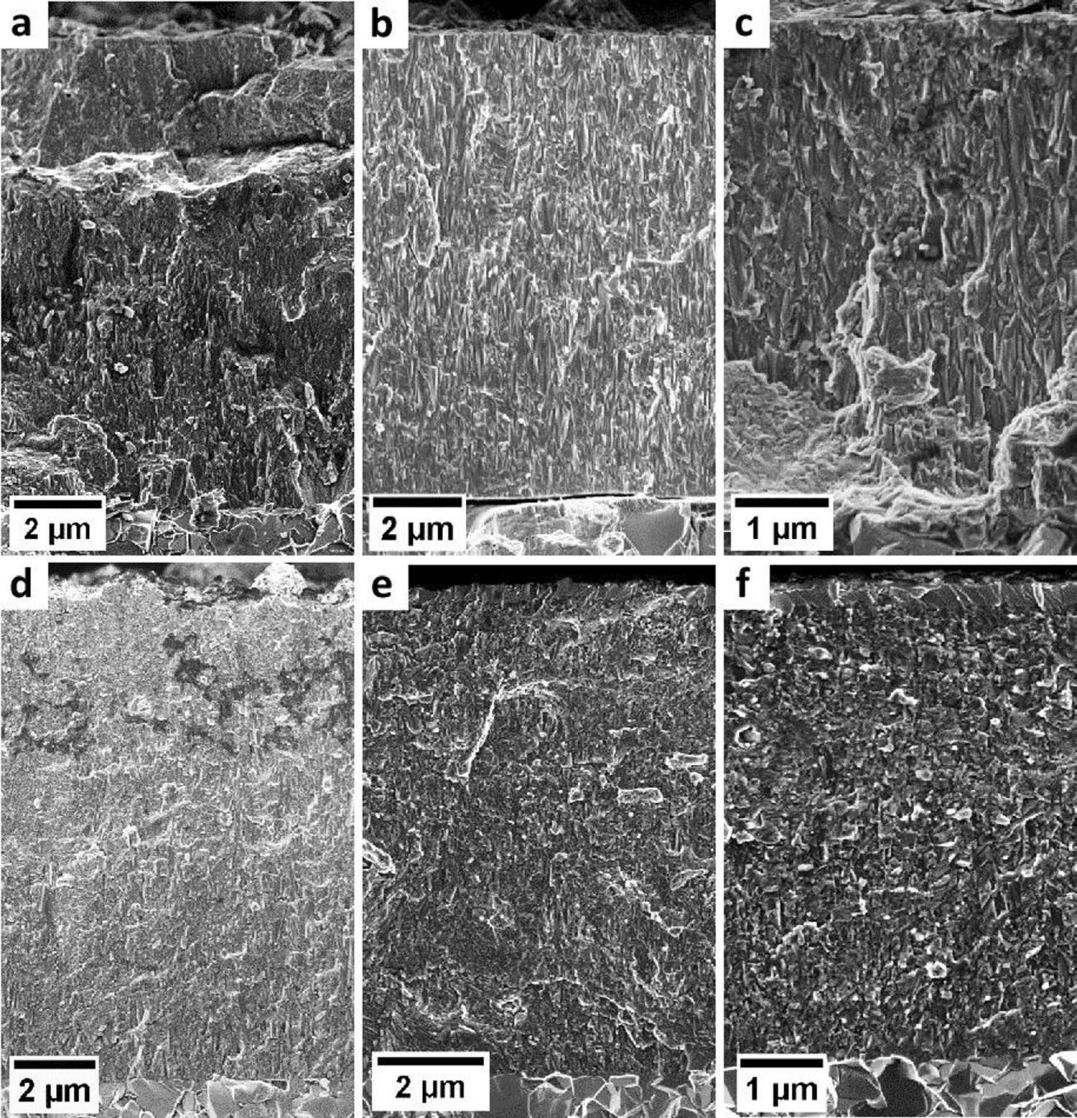
SEM micrographs of cross-sections of films A, B and C in as-deposited (**a**–**c**) and annealed (**d**–**f**) states, respectively.

Structural and chemical analysis performed by SEM on the film cross sections shows that the films possess (i) similar columnar grain-like microstructure in the as-deposited state irrespective of the deposition temperature (see Fig. 6a–c) and (ii) the same elemental composition within the detection limits of EDS (Table 2).

**Table 2:** Al and Cr content and Al/Cr ratio measured by EDS for thin films A, B and C.

	A	B	C
Al [%]	63.5 ± 1.0	63.5 ± 1.0	63.3 ± 1.0
Cr [%]	36.5 ± 1.0	36.5 ± 1.0	36.7 ± 1.0
Al/Cr ratio	1.74	1.74	1.72

After annealing, the cross-sectional microstructure changed to globular-like with many subgrains formed as a consequence of the decomposition of metastable c-AlCrN phase (section 4.2). Table 3 summarizes indentation hardness and modulus of all three AlCrN films investigated in this study.

**Table 3:** Hardness and indentation modulus for films A, B and C, in as-deposited and annealed state.

	as-deposited			annealed		
	A	B	C	A	B	C
H [GPa]	32.9 ± 3.5	32.9 ± 3.8	35.6 ± 2.7	28.8 ± 2.7	26.5 ± 2.0	28.7 ± 2.3
E_i_ [GPa]	449 ± 28	470 ± 32	484 ± 41	411 ± 28	425 ± 30	316 ± 19

It can be seen that both hardness and elastic modulus of the films in their as-deposited state increased with decreasing substrate temperature but are well comparable and much lower after annealing, which is associated with the changes of the crystal structure, morphology and residual stress state, as demonstrated by the results in Figs. 2–6.

## Discussion

The methodological novelty of this work resides in introducing the HT-HE-GIT XRD method, which was used to study the temperature dependent behaviour of Al_0.7_Cr_0.3_N films and WC-Co substrates and revealed the remarkable dependence of the film phase transformation temperature on the magnitude of the residual stress. Compared to other laboratory and synchrotron approaches reported in the literature, there is a twofold methodological advantage of the HT-HE-GIT XRD approach, namely (i) the collected 2D diffraction data provides a variety of experimental characteristics on phase composition, microstructure and strain evolution in film and substrate crystalline phases and (ii) the temperature-dependent values of CTEs from all film and substrate phases are evaluated experimentally and compared in order to quantify in-plane residual, thermal and intrinsic strains/stresses, which can be correlated with the actual film microstructure and phase evolution. Up to now, thin film lamellas of TiAlN^19^, ZrAlN^20^ and TiZrAlN^21^ were investigated in terms of phase and/or residual stress evolution at high temperatures. But, due to the reduced thickness of thin film lamellas, the residual stress state of the film on the lamella is expected to be different compared to the residual stress state of the film on a bulk substrate^29^. On the contrary, in the present approach, all thin film properties were measured on the bulk substrate, with little sample preparation effort. Furthermore, a minor disadvantage of the presented method is, that the film has to be positioned carefully with respect to the primary beam to optimize the film/substrate intensity ratio (c.f. Methods’ section) compared to probing a thin film lamella, where shading due to the substrate represents a minor complication. Additionally, (semi-)quantitative phase analysis during decomposition^17,18^ and the evaluation of thin film CTEs^36^ was in the past exclusively performed on thin film powders.

Therefore, the robust approach of HT-HE-GIT XRD available currently at the HEMS beamline of the Petra III light source in Hamburg provides temperature-dependent multi-parameter results and insights on the processes in the substrate and in the film simultaneously and allows to follow the evolution of phases, texture, CTEs, FWHMs (reflecting defects densities and grain sizes) and stresses variations, both in films and substrates, in real time.

Till now, the decomposition paths of the metastable CrAlN system has been mostly studied *in-situ* by differential scanning calorimetry combined with *ex-situ* structural analysis such as XRD or TEM or *ex-situ* by X-ray diffraction analysis of powders^5–7^.

In contrast, the HT-HE-GIT XRD method is well suitable to study the HT behaviour of metastable systems such as AlCrN, undergoing multiple-step phase decomposition. Although there are numerous reports on the decomposition paths of the AlCrN system^5–7^, the application of the HT-HE-GIT XRD method allows to get new insights especially into the stress development of individual phases, crystallographic relations between the precipitates and decomposed matrix and furthermore into the variation of thermal strains and thermal expansion of individual phases, all as a function of temperature by using a single setup in reasonable time.

The decomposition of the c-CrAlN system as a consequence of segregation of Al towards the GBs and subsequent formation of more thermodynamically stable Al-rich w-Al(Cr)N phases coexisting with Cr-rich c-Cr(Al)N proceeds in several steps, which can be described as follows:

$$\begin{array}{c}c-\mathrm{CrAlN}\to c-\mathrm{Cr}({\rm{Al}}){\rm{N}}+{\rm{w}}-{\rm{Al}}({\rm{Cr}}){\rm{N}}\to {\rm{c}}-{\rm{CrN}}\\ \,+{\rm{c}}-{\rm{Cr}}({\rm{Al}}){\rm{N}}+{\rm{w}}-{\rm{Al}}({\rm{Cr}}){\rm{N}}\to {\rm{w}}-{{\rm{Cr}}}_{2}{\rm{N}}\\ \,+{\rm{c}}-{\rm{Cr}}({\rm{Al}}){\rm{N}}+{\rm{w}}-{\rm{Al}}({\rm{Cr}}){\rm{N}}({\rm{lately}}\,{{\rm{Cr}}}_{2}{\rm{N}}\to {\rm{Cr}})\end{array}$$

This complete decomposition pathway observed during *in-situ* HT-HE-GIT XRD confirms in principal what is known from literature^5,6^ but is furthermore complemented by new important findings summarized hereafter.

During heating to *T*_S_, the lattice parameter d* continuously increases, which reflects the expansion of the crystal lattice. Since the CTE is a temperature-dependent physical quantity (see its non-linear increase with temperature in Fig. 3b), also the variation of the lattice parameter is not linear (Fig. 3a). On the contrary, as lattice defects generated during ion-assisted film growth and contributing to lattice distortion become mobile at temperatures higher than *T*_S_, the lattice parameter is reduced above ~500 °C and thus partially compensated for the increase of the lattice as a consequence of the lattice thermal expansion (visible as a decreased slope of the *d*(*T*) curve between 500 and 900 °C in Fig. 3a). The complementary FWHM analysis, which reflects the contribution of both the finite size of coherently diffracting domains and defect density, allows to identify the diffusion-induced defect recovery processes and crystallite size variations occurring during heating and cooling (Figs. 2g and 4b). No defect recovery of the c-CrAlN lattice or grain growth taking place at temperatures below *T*_S_ corresponds to insufficient energy delivered to the system below *T*_S_ to activate diffusion-driven relaxation and growth processes^28^. The analysis of lattice strain and its individual components revealed a continuous linear change of thermal strain ${\varepsilon }_{{\rm{th}}}^{{\rm{c}}}$ from tension to compression between RT and 900 °C as a consequence of a CTE mismatch of the film and substrate depicted in Fig. 3b, while the compressive intrinsic strain component ${\varepsilon }_{{\rm{i}}}^{{\rm{c}}}$ was constant to *T*_S_ followed by a gradual relaxation between *T*_S_ and the onset temperature of phase decomposition (Fig. 2c). The defect recovery above *T*_S_ is in good agreement with the development of the FWHM in this temperature range shown in Fig. 4b and is also reflected by the variation of the compressive residual strain ${\varepsilon }_{{\rm{r}}}^{{\rm{c}}}$ in Fig. 3c and residual stress evaluated in Figs. 2i and 4a, consisting of both intrinsic and thermal components.

Above ~700–900 °C, the ongoing relaxation of defects results in the formation of the w-Al(Cr)N phase at the GBs in all three investigated Al_0.7_Cr_0.3_N films, which is revealed in the phase plot in Fig. 2b by the appearance of the w-Al(Cr)N reflection and by a decrease of the lattice parameter d* (Fig. 3a), corresponding to the detriment of Al in the crystal lattice in the initial stage of the decomposition of c-AlCrN.

The segregated Al acts at the GBs as a nucleation site for the formation of w-Al(Cr)N crystalline nuclei, serving for further growth of the high Al-containing wurtzite phase. This interpretation was suggested by large FWHMs of the w-Al(Cr)N reflections with small intensity (Fig. 2c,d). Moreover, the c-Cr(Al)N crystallites obviously grew in the temperature range of 900–1100 °C, as indicated by a fast decrease of the FWHM shown in Figs. 2g and 4b.

The strength of the HT-HE-GIT XRD approach is also in the ability to reveal the nucleation and growth rate of the w-Al(Cr)N precipitates, which reached their maximum between 1000 and 1100 °C, as demonstrated by an increase of the first derivative of the peak intensity (Fig. 2d) and a decrease of the FWHM of the w-Al(Cr)N 100 reflection (Fig. 2g). Further decrease in the compressive strain above 1000 °C is associated with an interplay of several strain-reducing mechanisms associated with the ongoing decomposition of c-Cr(Al)N and CrN and phase softening. Moreover, the nitrogen loss and formation of the Cr_2_N phase associated with an increase of number of N vacancies results in a shrinkage of the crystal lattices (Figs. 2b and 3a), also accompanied by a reduction of the compressive strain. The capability of the HT-HE-GIT XRD method to detect the variations in ${\varepsilon }_{{\rm{i}}}^{{\rm{j}}}$, ${\varepsilon }_{{\rm{th}}}^{{\rm{j}}}$ and ${\varepsilon }_{{\rm{r}}}^{{\rm{j}}}$ nearly in the entire temperature range of the annealing experiment makes the method unique, especially because other parameters simultaneously detected during the experiment allow for interpretation of the origin of these variations and to reveal the ongoing processes. The onset temperature of decomposition of Cr_2_N at ~1080 °C (Fig. 2b–d and Supplementary Figs. S1b–d and S3b–d) is in good agreement with literature values^5,6,38^ of 1015–1140 °C.

During the holding segment at 1100 °C, the intensity increase of the w-Al(Cr)N and h-Cr_2_N reflections indicates further development of both phases (Fig. 2c), their volume increases at continuously decreasing rate (Fig. 2d). This indicates slowed-down diffusion of Al towards GBs of c-Cr(Al)N and nitrogen loss during the holding segment, which is furthermore corroborated by the almost constant lattice parameter d* of c-Cr(Al)N (Fig. 3a). A decrease in the FWHM of the w-Al(Cr)N crystallites indicates their continuous growth during the holding segment (Fig. 2g). The orientation of the c-Cr(Al)N and w-Al(Cr)N crystallites remains, however, unchanged (Fig. 2e,f).

During cooling from 1100 to 900 °C, the energy delivered to the system is obviously still sufficient for diffusion-driven structural variations, which are indicated by a further increase of the intensity of the w-Al(Cr)N and h-Cr_2_N reflections (Fig. 2c), even though at lower rates (Fig. 2d), revealing a decaying development of both phases. Since the film microstructure consists of three phases at this stage of the annealing experiment (c-Cr(Al)N, w-Al(Cr)N and h-Cr_2_N), which all differ in their CTEs, the thermal tensile strain continuously increase during cooling (note also different CTE of the WC-Co substrate). Remarkably, the residual stress of all phases exhibited rather the same values (Fig. 2i).

The HT-HE-XRD method allows identifying the role of deposition temperature on the development of film microstructure, individual stress components and stability of the c-CrAlN phase, which obviously differ with the increasing energy delivered to the system during the deposition process. While the microstructural changes with *T*_S_ were almost negligible, as demonstrated by almost identical SEM micrographs (Fig. 6) and FWHM values (Fig. 4b) of the films in the as-deposited state, their residual compressive stress state decreased with increasing temperature (Fig. 4a). Also the onset of defect recovery, indicating their thermal stability, is obviously proportional to the deposition temperature. This is evident from Fig. 4a by a deviation of *σ*_r_ from its thermo-elastic behaviour slightly above the varied *T*_S_. While the tensile thermal stress *σ*_th_ in the deposited state of the films increases with, and as a consequence of the deposition temperature, the compressive intrinsic stress *σ*_i_ decreased due to enhanced adatom mobility resulting in subsequent defect recovery occurring already during film growth (Fig. 5a). The development of the compressive residual stress to a different extent at various *T*_S_ results also in an increasing magnitude of the total stress recovery after the annealing experiment (Fig. 4a) indicating an obvious correlation between the residual stress state and the driving force for stress recovery during annealing. The deposition temperature also affects the thermal stability of intrinsic defects in the film microstructure. In agreement with other studies^30^, defect recovery was in all three cases detected above *T*_S_, which has to be exceeded to promote the diffusion processes and thus determines the defect stability. The residual stress state after the annealing was almost identical for all films (Fig. 4a). The difference of the FWHM of the films after the annealing experiment clearly showed that the c-Cr(Al)N crystallites grew much more than those of the films deposited at lower *T*_S_ (see smaller FWHM of the film A than that of B and C in Fig. 4b).

Since the elemental and phase composition of the films A, B and C (Table 2), their microstructure in terms of the crystallite size and texture (Fig. 2e and Supplementary Figs. S1e and S3e) as well as the FWHM were almost identical (the film texture moreover did not change during annealing), another effect controlling the thermal stability of the c-CrAlN phase needs to be considered. Whereas the onset temperature of phase decomposition was ranging between 698 and 914 °C from films A to C, respectively, the onset stress of phase decomposition was relatively constant between the magnitudes of −4560 and −3850 MPa (Table 1). Remarkably, the onset temperature of the phase decomposition lies within this range of about 600 MPa regardless of the thermomechanical history of the thin films.

The important role of the compressive residual stress magnitude on the decomposition of the metastable c-CrAlN solid solution may be related to the suppressed Al diffusion towards GBs. Also theoretical calculations are supporting the stabilization of the cubic structure at high compressive stresses estimated to −11.5 GPa to −16 GPa to stabilize the unstable c-AlN phase^39^. An analogy to this effect may be found in the stabilization of the cubic c-Cr_1-x_Al_x_N with respect to its composition. Theoretical calculations found that without compressive stresses, the cubic phase can be at 0 K stabilized up to x = 0.69, whereas in the presence of a hydrostatic compressive stress state of 4 GPa, the cubic phase can be stabilized up to x = 0.80^40^.

## Conclusions

*In-situ* high-temperature high-energy grazing incidence X-ray diffraction was used to analyse the (i) phases, (ii) texture, (iii) domain size, (iv) coefficients of thermal expansion and subsequently thermal strains, (v) residual and (vi) intrinsic stresses of three Al_0.7_Cr_0.3_N thin films on a cemented carbide bulk substrate. By using this novel approach, it was possible to comprehensively characterize the thermal stability of these thin films. The results revealed (i) a strain/stress controlled phase decomposition, where the decomposition onset temperature is predominantly dependent on the as-deposited in-plane residual stress magnitude, (ii) similar 〈111〉 fibre texture in the cubic phase and a combination of 〈100〉 and 〈110〉 in the hexagonal phase for all three investigated thin films and (iii) the measurement of CTE during heating and cooling for the substrate as well as the cubic and the hexagonal phase. The results demonstrate that the residual stress state plays a dominant role in enhancing the thermal stability of the metastable solid solution of c-AlCrN. Finally, it was shown, that the newly developed *in-situ* approach leads to a better understanding of the structural changes in metastable thin films at high thermal loads and can be applied as an powerful tool for further thin film design.

## Methods

### Thin film synthesis

The Al_0.7_Cr_0.3_N thin films were prepared by cathodic arc evaporation in a voestalpine eifeler-Vacotec alpha400P deposition system equipped with six Al_70_Cr_30_ cathodes operated at a cathode current of 100 A, substrate bias voltage U_B_ = −100 V and nitrogen pressure p_N2_ = 4 Pa. The deposition temperature varied from T_S, A_ = 475 °C, T_S, B_ = 400 °C to T_S, C_ = 325 °C for films denoted as A, B and C, respectively. The mirror-polished cemented carbide (WC, 6 wt.% Co) substrates of a dimension of 10 × 5 × 5 mm^3^ were mounted on the sample holder in one-fold-rotation at a cathode-to-substrate distance of ~100 mm and plasma cleaned prior deposition. Films A and B had a thickness of ~11 µm, while the film C had a reduced thickness of ~6 µm, as only 3 cathodes were used at the same deposition time in order to reduce the deposition temperature to 325 °C.

### Laboratory characterization

Cross-sectional characterization of the films was performed in a Zeiss LEO 1525 scanning electron microscope (SEM) with 3 kV accelerating voltage and an aperture of 20 µm. In order to reveal the elemental composition of the films, energy-dispersive X-ray spectroscopy analysis (EDS) was performed in SEM and quantified by built-in standards (Zeiss LEO 1525, Bruker Quantax, with 20 kV accelerating voltage and 60 µm aperture). Indentation modulus and hardness of the films were determined by means of nanoindentation (UMIS, Fischer-Cripps Laboratory Ltd.).

### *In-Situ* high-temperature synchrotron characterization

The *in-situ* HE-HT-GIT-XRD experiments were performed at the P07B beamline of the PETRA III synchrotron source in Hamburg (D) in transmission geometry, using a pencil X-ray beam with a size of 400 × 100 µm^2^ and an energy of 87.1 keV. The samples were mounted into a DIL 805 dilatometer (TA Instruments) with the surface aligned with respect to the primary beam at an incidence angle β of ~2 deg (Fig. 1). The incidence angle of ~2 deg was set to probe as much film volume as possible in transmission, whilst maintaining the intensity of substrate reflections at a minimum. The exact incidence angle and the position of the sample surface with respect to the primary beam are dependent on the individual sample and have to be repeatedly adjusted to an optimum before starting the *in-situ* HT-HE-GIT experiment.

The thermal cycle included heating to 1100 °C at a rate of 1 K/s followed by a holding segment of 300 s at the maximum temperature (both in vacuum at p_total_ < 10^−2^ mbar) and subsequent cooling to RT at a rate of ~1 K/s, controlled by the Ar flow through the dilatometer chamber. Two dimensional (2D) X-ray diffraction patterns were recorded continuously using a Perkin-Elmer detector with a pixel size of 200 × 200 µm² and an exposure time of ~25 s per frame (Fig. 1). The temperature for each diffraction pattern exposure was recorded with a Type S thermocouple welded to the sample surface with a resolution of ~0.5 °C. The detector calibration was performed using a LaB_6_-powder and the Fit2D software package^41^. The sample-to-detector distance, the detector tilt and the rotation angle of the tilt plane of 1949.4 mm, 0.0895 deg and 69.453 deg were evaluated, respectively. The beam centre on the detector was evaluated from the lower left corner 1031.246 and 978.412 pixels in horizontal and vertical direction. The 2D data evaluation was performed using the pyFAI software package^42^.

## Supplementary Material

Supplementary Material

## Data Availability

The authors declare that all data are available on request.
