# Supplementary material for: Stress-controlled decomposition routes in cubic AlCrN films assessed by in-situ high-temperature high-energy grazing incidence transmission X-ray diffraction
Source: Sci Rep. 2019 Dec 2;9:18027. doi: 10.1038/s41598-019-54307-7 (PMC6888894; doi:10.1038/s41598-019-54307-7)
Supplement: Supplementary file 1 — Supplementary Material [file 41598_2019_54307_MOESM1_ESM.pdf]

Stress-controlled decomposition routes in cubic AlCrN films assessed by in-situ high-temperature high-energy grazing incidence transmission X-ray diffraction

M. Meindlhumer[1], S. Klima[1], N. Jäger[1], A. Stark[2], H. Hruby[3], C. Mitterer[4], J. Keckes[4], R. Daniel[1]

[1]Christian Doppler Laboratory for Advanced Synthesis of Novel Multifunctional Coatings at the Department of Materials Science, Montanuniversität Leoben, Leoben, Austria

[2]Helmholtz-Zentrum Geesthacht, Centre for Materials and Coastal Research, Geesthacht, Germany

[3] voestalpine eifeler Vacotec GmbH, Düsseldorf, Germany

[4]Department of Materials Science, Montanuniversität Leoben, Leoben, Austria

Supplementary Figures:

Supplementary Figure S1:

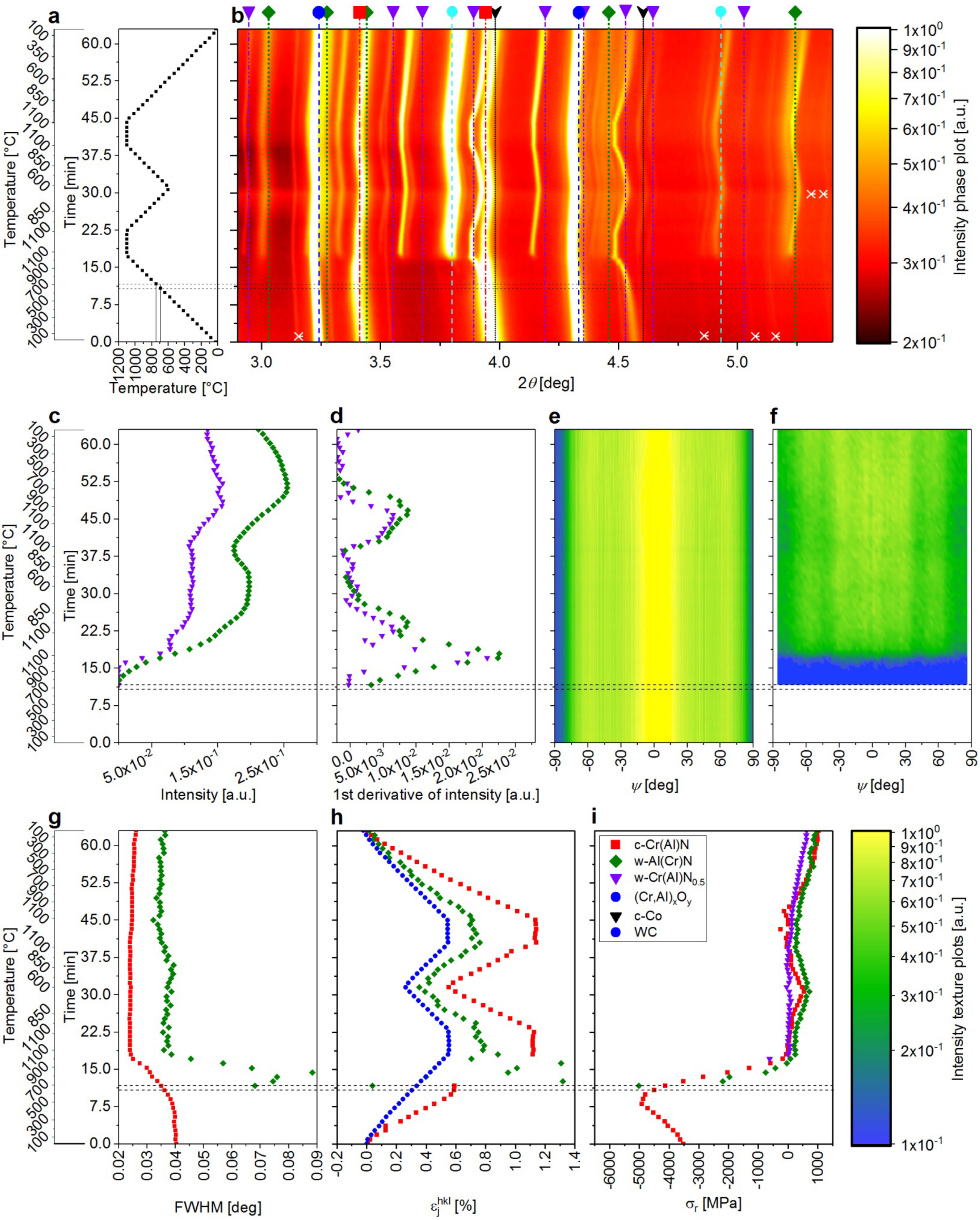

Suppl. Fig. S1. The applied temperature cycle (a) and the experimental data of the AlCrN film A, phase plot with indicated diffraction angles for tabulated particular phases, where the white crosses indicate additional diffraction peaks representing 2nd order diffraction due to the presence of the second harmonic's wavelength in the primary beam (b), evolution of intensity(c) and the 1st derivative of the intensity (d) of w-Al(Cr)N 100 (green) and h-Cr_2N 100 (violet) reflections, the texture plot for c-Cr(Al)N 111 reflection indicating <111> fibre texture (e), texture plot for w-Al(Cr)N 100 reflection indicating overlapping <100> and <110> fibre texture (f), evolution of FWHM of c-Cr(Al)N 111 (red) and w-Al(Cr)N 100 (green) reflections (g), thermal expansion of c-Cr(Al)N (red, evaluated from the 200 reflection), w-Al(Cr)N (green, 100 reflection) and WC substrate (blue) (h) and residual stress evaluated from the c-Cr(Al)N 200 (red), w-Al(Cr)N 100 (green) and h-Cr_2N 100 (violet) reflections (i).

Supplementary Figure S2:

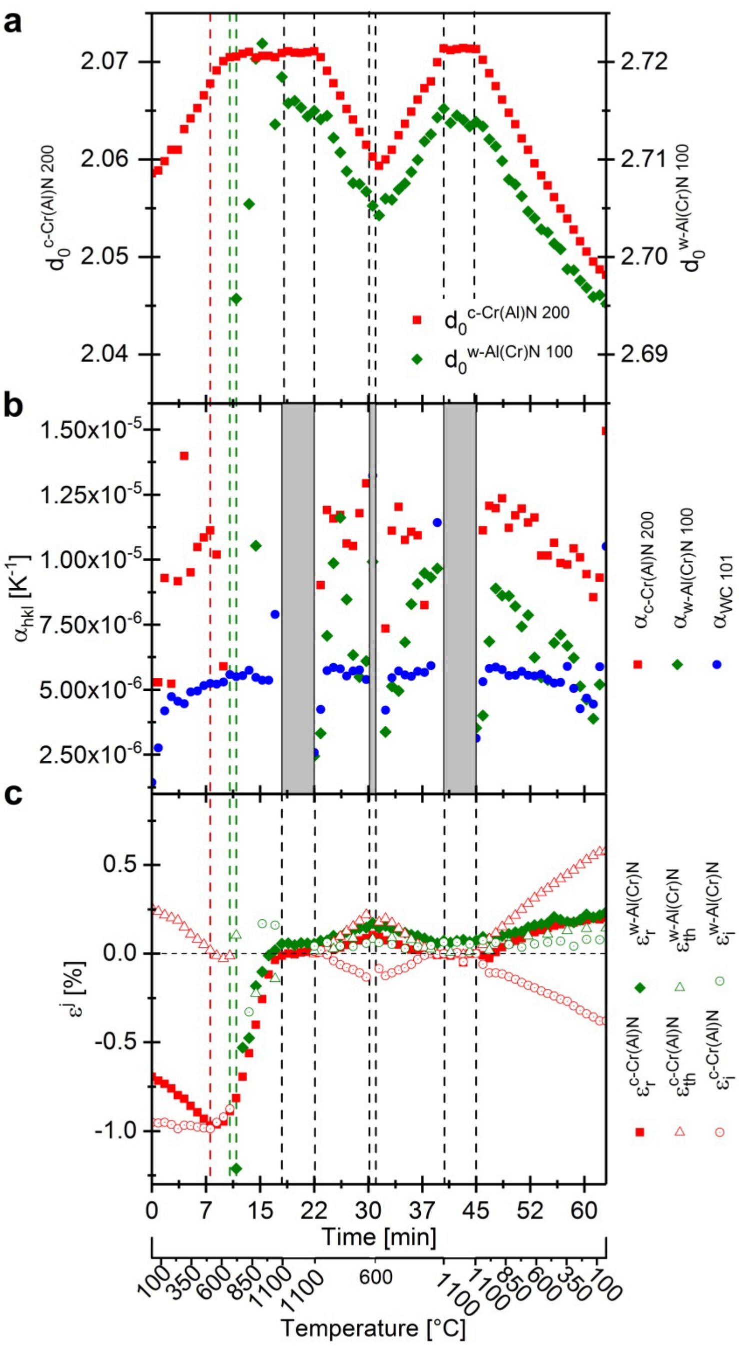

Suppl. Fig. S2. Experimental assessment for film A deposited at 475°C: development of the unstrained lattice parameter $d_0(T)$ of the c-Cr(Al)N and the w-Al(Cr)N phase, respectively (a), the thermal expansion coefficient calculated for the individual reflections (b) and the evolution of thermal, intrinsic and residual strain over the temperature cycle for c-Cr(Al)N and w-Al(Cr)N, respectively (c). The vertical red dashed line represents the deposition temperature, the green dashed line the onset of the phase decomposition and the vertical black segmented lines the beginning and the end of the holding segments.

Supplementary Figure S3:

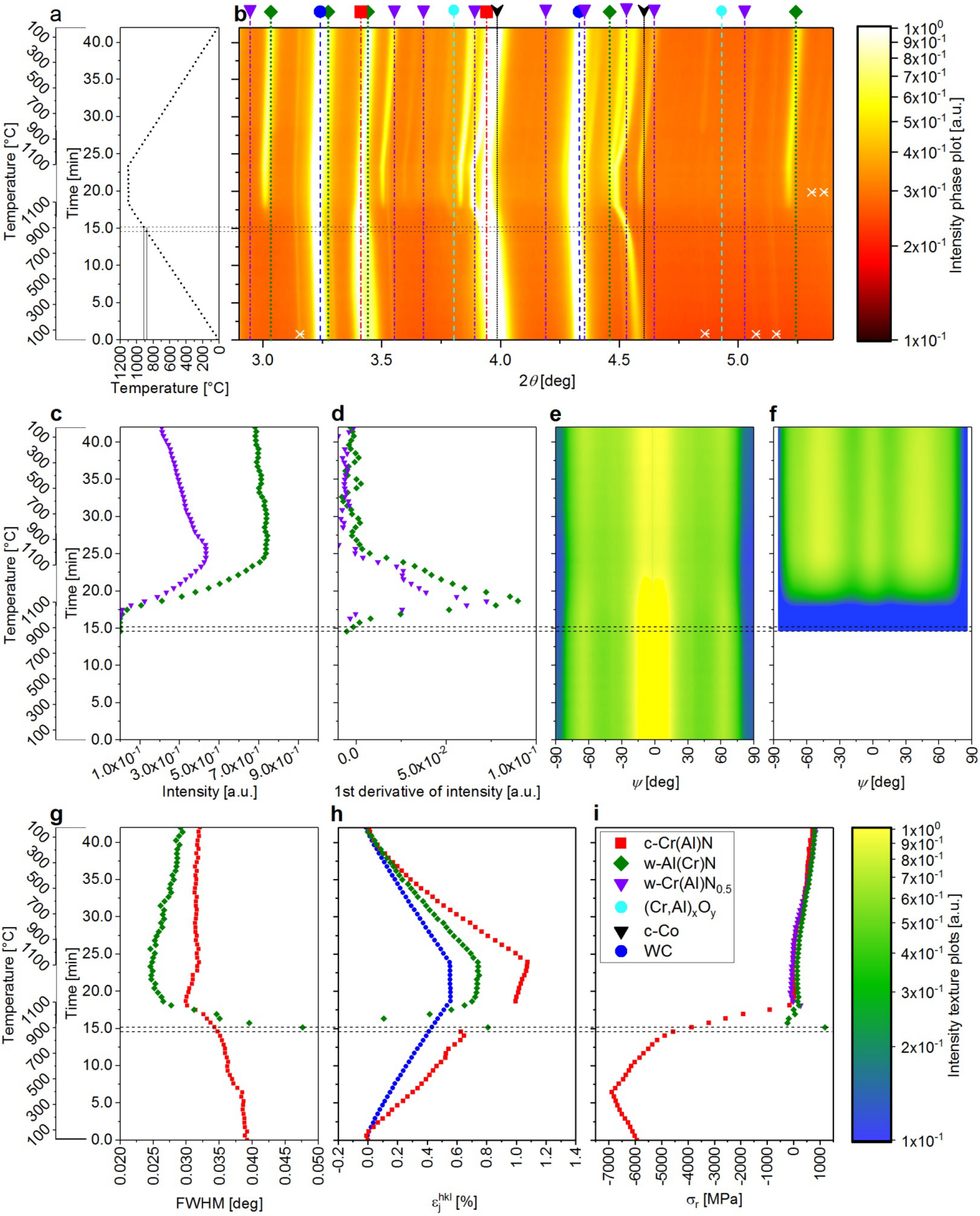

Suppl. Fig. S3. The applied temperature cycle (a) and the experimental data of the AlCrN film C, phase plot with indicated diffraction angles for tabulated particular phases, where the white crosses indicate additional diffraction peaks representing 2nd order diffraction due to the presence of the second harmonic's wavelength in the primary beam (b), evolution of intensity (c) and the 1st derivative of the intensity (d) of w-Al(Cr)N 100 (green) and h-Cr2N 100 (violet) reflections, the texture plot for c-Cr(Al)N 111 reflection indicating <111> fibre texture (e), texture plot for w-Al(Cr)N 100 reflection indicating overlapping <100> and <110> fibre texture (f), evolution of FWHM of c-Cr(Al)N 111 (red) and w-Al(Cr)N 100 (green) reflections (g), thermal expansion of c-Cr(Al)N (red, evaluated from the 200 reflection), w-Al(Cr)N (green, 100 reflection) and WC substrate (blue) (h) and residual stress evaluated from the c-Cr(Al)N 200 (red), w-Al(Cr)N 100 (green) and h-Cr_2N 100 (violet) reflections (i).

Supplementary Figure S4:

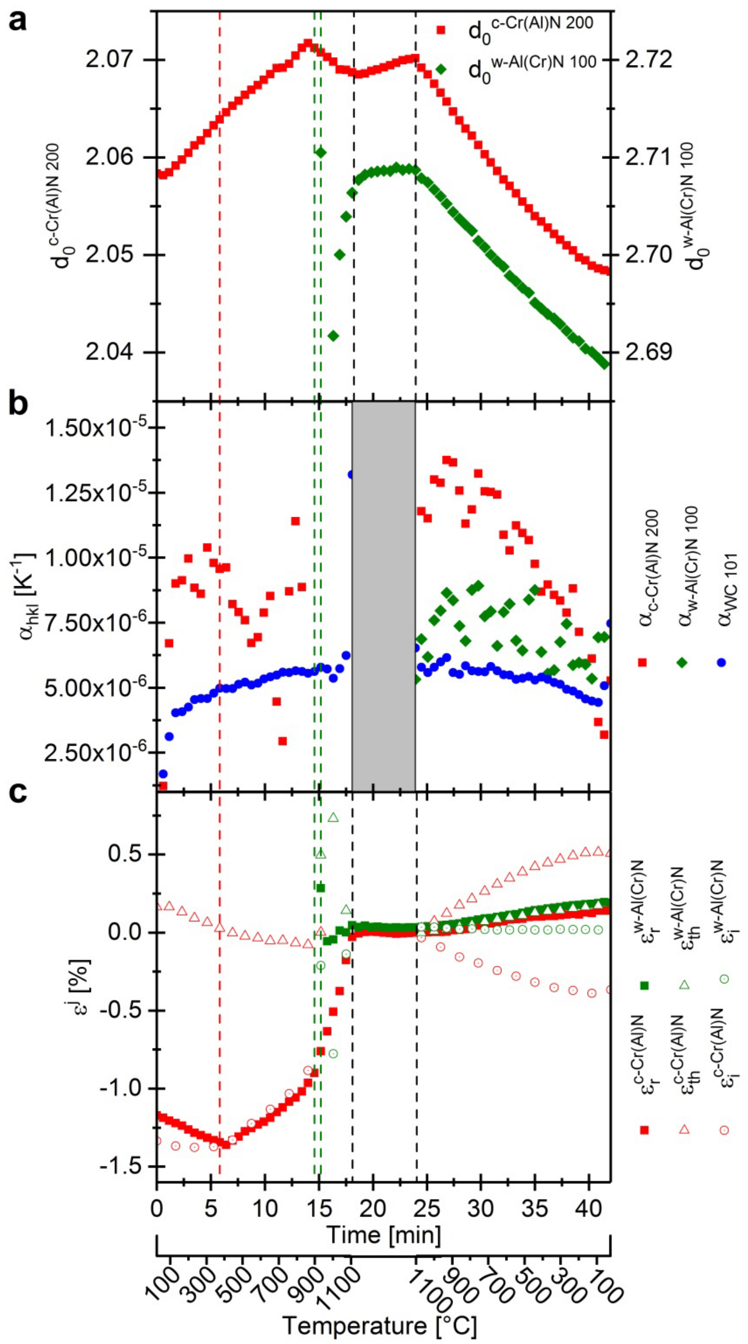

Suppl. Fig. S4. Experimental assessment for film C deposited at 325°C: development of the unstrained lattice parameter $d_0(T)$ of the c-Cr(Al)N and the w-Al(Cr)N phase, respectively (a), the thermal expansion coefficient calculated for the individual reflections (b) and the evolution of thermal, intrinsic and residual strain over the temperature cycle for c-Cr(Al)N and w-Al(Cr)N, respectively (c). The vertical red dashed line represents the deposition temperature, the green dashed line the onset of the phase decomposition and the vertical black segmented lines the beginning and the end of the holding segments.
